# Under-reported complications related to BMP use in spine surgery

**DOI:** 10.3109/17453674.2011.623576

**Published:** 2011-11-24

**Authors:** Per Aspenberg

**Affiliations:** Department of Orthopaedics, IKE, Faculty of Health Sciences, Linköping University, Swedenper.aspenberg@liu.se

A recent review article and editorial in the Spine Journal shows that in 13 industry-sponsored trials of recombinant BMP-2 for spine applications in a total of 780 patients, not a single drug-related complication was reported (Carragee et al. 2011a, b). This contrasts with FDA databases and unsponsored case series showing several drug-related problems, such as dramatic local bone resorption or local swelling leading to airway obstruction. The review and the subsequent jaw-dropping correspondence leaves little doubt that several of the industry-sponsored papers were deliberately deceitful.

New methods commonly appear to work better in the hands of the inventor. This may be related to higher competence, e.g. by more careful patient selection. However, the risk of self-delusion is of course high, when a lot of prestige is involved (as for the academic inventor) or when millions of dollars relate the investigator to the industry, as in many of the studies of BMP-2 in spine surgery. In contrast to spine studies, unfavorable results with BMP-2 in tibial fractures seem to have been meticulously reported (Aro et al. 2011).

The BMP-2 spine affair illustrates the folly of grading studies for level of evidence, based on standardized criteria. The “level 1” trials may have been misleading, but the complication rates in “level 3” case series should have given reasons for thinking twice. There is no way to make good clinical decisions based on standard criteria without critical thinking. But, alas, thinking clearly is so difficult. One of the problems is that we like good news, whereas reading about obstacles and complications makes no one happy (except those of us who enjoy a trace of academic sadism). Actually, Acta Orthopedica pioneered the reporting of BMP-related problems: one of the first case series (perhaps the very first) with a BMP for cervical spine surgery was published in Acta Orthopedica already in 1999 (Jeppsson et al.). The series had to be stopped due to a complete lack of effect in 3 of the first 4 patients. This did not reduce the general enthusiasm for BMPs, and the senior author went on to a moderately successful trial on lumbar spine (it was me). Acta Orthopedica also published very early animal work showing that BMPs could actually reduce net bone formation (Jeppsson and Aspenberg 1996, Jeppsson et al. 1999). Again, this passed without much notice. Later, during the first years of BMP trials, many rumors circulated within the “BMP community” about problems with local swelling, and the first case of resorption of a vertebral body was published as early as in 1999 (Laursen et al.). Still, we all hoped that this was part of a learning curve, and we were afraid to throw out the baby with the bathwater. After all, John Charnely's first 300 cases with hip replacements largely failed, but in the end his technique became one of the greatest achievements in orthopedics—ever.

Current thinking mainly suggests that the problem of investigator bias could be solved by publishing details of the authors' economic ties to industry. The underlying thought is that economic support corrupts. But does it always? Should a study with good financial support automatically be regarded as less reliable? There may be a difference between studies initiated and performed by the industry and studies initiated by academic researchers but funded by the industry. In the latter case, an author may have a stronger position in discussions with the company's marketing department. Economic ties may carry a higher risk of bias, but after all, a wish for academic prestige may be an even more common cause of scientific misconduct than economic profit. Publishing of economic ties might be a good idea, but it is more important that authors know that if they deceivingly omit serious complications, it may be the last paper they are ever able to publish.

However, the responsibility for possible over-use of BMP-2 lies not only with sponsors, authors, and editors but also with readers and the orthopedic community as a whole. The early warnings of problems related to BMPs, and the published complications, were not sufficiently discussed at the time, especially not at the many industry-sponsored satellite sessions held at academic meetings. Such meetings might be more responsible for the spread of biased opinions than journal articles. Again, the orthopedic decision-maker has to rise above the level of just keeping a finger in the air to feel the latest trends. He or she must be an intellectual who doesn't rush to introduce new techniques, but reads and thinks critically. This might become increasingly difficult now in the Nordic countries too, as private care and market thinking are gaining ground.
